# Cardiovascular Risk Factors and Hemodynamic Measures as Determinants of Increased Arterial Stiffness Following Surgical Aortic Valve Replacement

**DOI:** 10.3389/fcvm.2021.754371

**Published:** 2021-12-08

**Authors:** Oscar Plunde, Anders Franco-Cereceda, Magnus Bäck

**Affiliations:** ^1^Unit of Cardiovascular Medicine, Department of Medicine Solna, Karolinska Institutet, Stockholm, Sweden; ^2^Theme Heart and Vessels, Division of Valvular and Coronary Disease, Karolinska University Hospital, Stockholm, Sweden; ^3^Cardiothoracic Surgery Unit, Department of Molecular Medicine and Surgery, Karolinska Institutet, Stockholm, Sweden

**Keywords:** aortic stiffness, arterial stiffness, aortic stenosis, aortic, aortic regurgitation, surgical aortic valve replacement

## Abstract

Valvular and arterial function are tightly intertwined, both in terms of structural changes and hemodynamics. While proximal valvulo-vascular coupling contributes to the cardiovascular consequences of aortic stenosis, less is known on how peripheral arterial stiffness relates to aortic valve disease. Previous studies have shown conflicting results regarding the impact of aortic valve replacement on arterial stiffness. The aim of the present study was therefore to determine predictors of arterial stiffness in patients with and without aortic valve disease undergoing cardiac surgery. Cardio ankle vascular index (CAVI) and carotid femoral pulse wave velocity (cfPWV) were measured to determine arterial stiffness the day before and 3 days after surgery for either ascending aortic or aortic valve disease. Stratification on indication for surgery revealed that CAVI was significantly lower in patients with aortic valve stenosis (*n* = 45) and aortic valve regurgitation (n=30) compared with those with isolated ascending aortic dilatation (*n* = 13). After surgery, a significant increased CAVI was observed in aortic stenosis (median 1.34, IQR 0.74–2.26, *p* < 0.001) and regurgitation (median 1.04, IQR 0.01–1.49, *p* = 0.003) patients while cfPWV was not significantly changed. Age, diabetes, low body mass index, low pre-operative CAVI, as well as changes in ejection time were independently associated with increased CAVI after surgery. The results of the present study suggest aortic valve disease as cause of underestimation of arterial stiffness when including peripheral segments. We report cardiovascular risk factors and pinpoint the hemodynamic aspect ejection time to be associated with increased CAVI after aortic valve surgery.

## Introduction

Aortic valve stenosis (AVS), characterized by fibrosis and calcification of the aortic valve is preceded by sclerosis (AVSc) without significant hemodynamic consequences. Progression to AVS leads to reduced valve opening and eventually significant left ventricle (LV) outflow obstruction. AVS is the most common valvulopathy requiring intervention, which can be performed either through transcatheter aortic valve implantation (TAVI) or by surgical aortic valve replacement (SAVR). The pathophysiological processes in AVS share many features with atherosclerosis which is associated with arterial stiffness (1). A connection between arterial stiffness and AVS is supported by the association of aortic valve calcification and arterial stiffness (2), measured as either increased carotid femoral pulse wave velocity (cfPWV) in patients with aortic stenosis (3) or a higher cardio ankle vascular index (CAVI) (4) in patients with AVSc compared with controls (5).

A close valvulo-arterial interplay has been established as an important factor determining the LV load in AVS (6). In particular, an increased valvulo-arterial impedance is associated with poor outcome in patients with AVS (7). Furthermore, a high PWV prior to TAVI predicts mortality (8), further reinforcing the importance of the valvulo-arterial interplay. It can hence be anticipated that the arterial function is altered after aortic valve intervention. Indeed, arterial function measured after AVR with different approaches including invasive pressure-wire techniques (9), aortic stiffness index (ASi) (10) and ascending aortic PWV using cardiac magnetic resonance imaging (cMRI) (11) indicate increased measures of arterial stiffness. Although, other studies have generated inconsistent results with unchanged arterial function after AVR measured with, non-invasive augmentation index (12), ASi (13), and cMRI in TAVI patients (11). In contrast to some of the above-mentioned methods, cfPWV is a direct, non-invasive and validated method to determine aortic stiffness. It includes the aorta and is based on the propagation of the pulse wave generated by the LV, in which a stiffer artery yields a faster pulse wave. To our knowledge, only 3 previous studies have determined cfPWV before and after AVR of which 2 indicated increased cfPWV (14, 15) after AVR and 1 suggested unchanged cfPWV (16).

While cfPWV is gold standard (17, 18) in non-invasive measurement of aortic stiffness, it is largely dependent on blood pressure (19), which may exhibit considerable changes after AVR. In contrast, CAVI, which measures the arterial stiffness from a larger proportion of the arterial tree including peripheral segments, is less dependent on blood pressure (20) and has low interobserver variation (21). To our knowledge, no previous study has assessed arterial stiffness with CAVI in AVS patients and the relationship between peripheral arterial stiffness and aortic valve disease remains to be deciphered.

Therefore, the aims of this study were to determine (i) arterial and aortic stiffness before and after cardiac surgery (ii) differences between patients undergoing surgery for either aortic valve or ascending aortic pathology, and (iii) the predictors of arterial and aortic stiffness and their changes in each diagnosis group.

## Materials and Methods

### Patients

Patients referred for surgical intervention due to AVS, aortic regurgitation or ascending aortic dilatation were prospectively included as part of the DAVAACA (Disease of the Aortic Valve Ascending Aorta and Coronary Arteries) study. DAVAACA is an ongoing single-center cohort study that includes patients undergoing elective open-heart, aortic and/or aortic valve surgery with or without concomitant coronary artery bypass grafting (22). Patients in this present study were recruited between 2017 and 2019. All participants gave written informed consent. The study was approved by the local ethics committee “Regionala etikprövningsnämnden i Stockholm” (2012/1633-31/4 with amendment 2016/2346-32) and conducted in agreement with the declaration of Helsinki. Of 108 patients screened and/or examined for this study, 88 had conclusive measures at baseline and 68 post-surgery (Supplementary Material 1). Reasons for exclusion were lower limb amputation, ankle brachial index <0.9, technical issues and atrial fibrillation. Technical issues included inability to get sufficient pulse wave registration for carotid femoral pulse wave velocity (cfPWV, *n* = 3) and inadequate phonocardiogram (PCG)-registration for cardio ankle vascular index (CAVI). Only patients that completed CAVI and cfPWV were included. The patients were stratified based on main indication for surgery: AVS, aortic regurgitation (AR) or ascending aortic dilatation (AAD) without AR. This stratification was rationalized by (i) previous studies indicate post-AVR changes in arterial stiffness in AR and AVS and (ii) to able to have a control group free from aortic valve disease. Data from electronic medical records were captured from the pre-operative day. Left ventricular ejection fraction (EF) was categorized as ≥50% and <50% to define normal and decreased EF (23), respectively. Information on EF was collected from the echocardiography report from latest available pre-operative and the first available post-operative (median post-operative day 3, IQR 2-6) examinations performed as part of clinical routine.

### Cardio Ankle Vascular Index and Carotid Femoral Pulse Wave Velocity Measures

All measurements were made in a private patient room after 10 min rest, in supine position at normal room temperature, the day prior to scheduled surgery (median 1 day, IQR 1–1). The post-operative measurements were performed in the same environment 3 days after surgery (median 3 days, IQR 3–3). Patients remained on their habitual anti-hypertensive treatment, which was not routinely paused the day before surgery.

CAVI, right brachial blood pressure and heart rate (HR) were measured and mean arterial pressure (MAP) and pulse pressure (PP) were calculated using VaSera-1500 (Fukuda, Denshi). CAVI aim to capture the intrinsically arterial stiffness from the beginning of the aorta to the ankle and was developed in 2004 (4), inspired by the β-stiffness (24) index and the Bramwell Hill Formula (25). It is obtained from heart-ankle PWV by the equation:


$$\begin{array}{l} CAVI=a\left\{\frac{2\rho }{PP}\;x\;\left(\ln\frac{SBP}{DBP}\right)\;x\;{\left(\frac{L}{tba+tb}\right)}^{2}\right\}\;x\;b \end{array}$$

where ρ = blood density (1.05), PP =pulse pressure, SBP and DBP = systolic and diastolic blood pressure respectively, L = the length from the aortic valve to the ankle, tba = the difference between time to start of the brachial pulse and time to ankle pulse and tb = time from aortic valve to the brachial pulse (measured from the second heart sound to the dicrotic noth at the brachial pulse wave form). In addition, brachial ankle pulse wave velocity (baPWV) (26, 27) was estimated by the formula (0.5934 × height (cm) + 14.4724)/tba. Only measurements deemed acceptable by the device (+ or ++) were included. The average between right and left CAVI was used.

Upstroke time (UT) denotes the time from the initial notch of the pulse wave to the peak and ejection time (ET) depicts time of blood flow across the aortic valve and were both were monitored using VaSera-1500. Since ET is dependent on heart rate, heart rate corrected ET (ETc) was used and calculated as ET(ms)/RR-interval(ms).

The right brachial blood pressure measured with VaSera-1500 was used.

Aortic stiffness was assessed with cfPWV using applanation tonometry (Sphygmocor, AtCor Medical, Sydney, Australia) directly after the CAVI measurement. This method yields an estimated PWV in the entire aorta although it should be acknowledged that the measuring sites are indeed in 2 peripheral sites. cfPWV is calculated by dividing time it takes for the pulse wave to travel a distance (d). The carotid-femoral distance was determined by measuring distance from the suprasternal notch (SN) to the place for obtaining the carotid pulse (d1) and from the SN to the place for obtaining the femoral pulse (d2). The final distance was calculated by the manufacturer program by d2–d1. The time to detection of the pulse wave was registered with a three led electrocardiogram using the foot-to-foot method. The beginning of the wave was identified with intersecting tangent algorithms.

### Statistical Methods

Categorical data are presented as numbers and per cent and continuous data as either median and IQR (25th−75th percentile) or mean (standard deviation). Kruskal-Wallis or analysis of variance (ANOVA) was performed when comparing continuous data between groups as appropriate, with Bonferroni adjusted *post-hoc* test when applicable and an alpha level of 0.05 was chosen. Fisher's exact test was used when comparing categorical data between groups. ANCOVA was performed to assess differences between diagnosis groups while adjusting for confounders. Included covariates in the ANCOVA were based on physiological relevance and univariate Pearson correlations. Multi-collinearity and overfitting were avoided. Age, sex, height, HR, MAP, eGFR, diabetes, CRP were included as covariates in the model for the baseline and post-surgery cross-sectional comparisons. Repeated measures ANCOVA was used to compare pre- and post-operative CAVI and cfPWV while controlling for the change in MAP and HR.

A backward stepwise regression was used to find independent predictors of the observed increase in CAVI following surgical valve intervention. Age, sex, ΔHR, ΔETc, ΔMAP, baseline CAVI, BMI, diabetes, eGFR, and AVS were removed from the model with a backward method to avoid overfitting and potential multicollinearity problem.

For all general linear models, outliers were removed prior to analysis and if normal distribution was not met, the data was log2-transformed resulting in normality which was tested with Shapiro-Wilk test. Cook's distance was used to control for outliers and a value <1 was tolerated. The normality of residuals was assessed by a P-P plot and homoscedasticity by Levene's test or a plot with regression standardized predicted values and regression standardized residuals. Standardized residuals were always >-3 to <3. Non-multicollinearity was assured with variance inflation factor (VIF) <5. Homogeneity of regression slopes in ANCOVA was assured by a non-significant interaction between the covariates and the diagnosis groups. SPSS 25.0 for Mac (IBM Corp., Armonk, NY) and R version 4.0.3 were used for statistical analyses.

## Results

### Arterial and Aortic Stiffness Associations

The baseline characteristics of the 88 included patients are shown in Table 1 and the flow chart for patient inclusion is shown in Supplementary Material 1. In the AAD group, 1 Marfan patient was included and all other were either degenerative dilatation or associated with BAV. All AAD patients received graft-procedures and 4 patients in the AR group also received a freestyle root.

**Table 1:** Baseline patients characteristics.

	**Aortic stenosis**		**Aortic regurgitation**		**Ascending aortic dilatation**		
	***N***	**Median (IQRs) or No. (%)**	***N***	**Median (IQRs) or No. (%)**	***N***	**Median (IQRs) or No. (%)**	***p*-value**
Age (years)	45	69 (64–74)^*^	30	59 (51–69)	13	63 (49–71)	0.007
Male sex	45	30 (67%)	30	24 (80%)	13	9 (69%)	0.44
BMI (kg/m^2^)	45	27.5 (24.2–31.1)	30	26.1 (23.8–30.7)	13	24.8 (23.5–28.4)	0.33
Current smoker	41	7 (17%)	26	3 (12%)	10	1 (10%)	0.83
TAV	45	20 (44%)	26	16 (55%)	13	9 (69%)	0.30
**Surgical procedures**							
CABG	45	8 (18%)	30	1 (3%)	13	0 (0%)	0.066
CAD	45	12 (27%)	30	2 (7%)	13	1 (8%)	0.049
AAD	45	9 (20%)	30	23 (77%)	13	13 (100%)	<0.001
AR	45	5 (10%)	30	30 (100%)	13	0 (0%)	<0.001
AVR	45	45 (100%)	30	26 (87%)	13	0 (0%)	<0.001
Mechanical prosthesis	45	10 (22%)	26	7 (27%)	13	0	0.77
**Medications**							
ASA	45	23 (51%)	30	9 (30%)	13	3 (23%)	0.084
β-Blocker	45	15 (33%)	30	12 (40%)	13	5 (39%)	0.83
ACEi/ARB	45	17 (38%)	30	16 (53%)	13	9 (69%)	0.10
Ca-blocker	45	9 (20%)	30	6 (20%)	13	6 (46%)	0.12
Diuretics	45	10 (22%)	30	6 (20%)	13	1 (8%)	0.62
Lipid Lowering	45	26 (58%)	30	8 (27%)	13	4 (31%)	0.018
**Comorbidities**							
Diabetes	45	8 (18%)	30	1 (3%)	13	0 (0%)	0.066
Hypertension	45	19 (42%)	30	12 (40%)	13	7 (54%)	0.69
**Laboratory parameters**							
Hb (g/L)	45	136 (129–148)	30	142 (136–151)	13	144 (140–150)	0.047
CRP (mg/L)	45	1 (0.9–2)	30	1.0 (0.9–2.3)	13	1 (0.9–2.5)	0.98
Calcium (mmol/L)	45	2.37 (2.30–2.43)	29	2.37 (2.33–2.41)	12	2.38 (2.31–2.47)	0.50
eGFR (1.73/ml/m^2^)	45	72 (59–81)	30	73 (66–90)	13	69 (56–91)	0.61

*P-values from analysis of variance (ANOVA), Kruskal-Wallis test or Fisher's Exact test as appropriate. BMI, body mass index; TAV, tricuspid aortic valve; CABG, coronary artery bypass surgery; CAD, coronary artery disease; AAD, ascending aortic dilatation; AR, aortic regurgitation; AVR, aortic valve replacement; ASA, acetylic salicylic acid 75 mg; ACEi/ARB, angiotensin converter enzyme-inhibitor/angiotensin receptor blocker; CRP, C-reactive protein; eGFR, estimated glomerular filtration rate*.

* *Indicate Bonferroni adjusted p-value < 0.05 compared with AR-group*.

The different measures of arterial and aortic stiffness, CAVI, brachial ankle pulse wave velocity (baPWV) and cfPWV were significantly associated with each other and between the pre- and post-operative measures (Supplementary Materials 2, 3). In univariate analysis, CAVI was associated with age and mean arterial pressure (MAP) and inversely with estimated glomerular filtration rate (eGFR) pre-operatively (Supplementary Material 2). In contrast, post-operative CAVI was associated with age, diabetes and hypertension and inversely with body mass index (BMI), and heart rate (HR) (Supplementary Material 2). For cfPWV, there were significant univariate associations with age, MAP, hypertension, C-reactive protein (CRP) and inverse associations with eGFR and male sex (Supplementary Material 2). Post-operatively, cfPWV showed a stronger correlation with age, HR and CRP and was no longer significantly associated with sex, MAP, hypertension or eGFR. In line with CAVI and cfPWV, Age, hypertension, eGFR and MAP were all associated with baPWV before and after surgery (Supplementary Material 3).

After stratification based on main indication for surgery: AVS, aortic regurgitation (AR) or ascending aortic dilatation (AAD) without AR, the overall trend for the differences in pre-operative CAVI between the groups was at the limit of significance (Table 2), despite a significant age difference. The baPWV, measured concurrently with CAVI, was significantly higher in the AAD group compared to AR group. For cfPWV, the lowest measures were observed in the AR group, followed by AVS and with the highest cfPWV in the AAD group, although not reaching statistical significance for the overall trend (Table 2).

**Table 2:** Pre-operative hemodynamic and stiffness parameters.

	**Aortic stenosis**		**Aortic regurgitation**		**Ascending aortic dilatation**		
	***N***	**Median (IQRs) or No. (%)**	***N***	**Median (IQRs) or No. (%)**	***N***	**Median (IQRs) or No. (%)**	***p*-value**
cfPWV (m/s)	45	8.0 (7.2–9.7)	30	7.1 (6.0–9.2)	13	8.5 (6.2–10.8)	0.067
CAVI	45	7.85 (7.07–8.59)	30	7.35 (6.59–8.46)	13	8.65 (7.23–10.17)	0.053
baPWV (cm/s)	43	1,292 (1,190–1,473)	29	1,220 (1,112–1,380)^†^	12	1,585 (1,361–1,896)	0.008
HR (bpm)	45	66 (58–74)	30	63 (57–70)	13	60 (57–69)	0.47
Systolic BP (mmHg)	45	139 (132–153)	30	147 (134–156)	13	144 (136–155)	0.42
Diastolic BP (mmHg)	45	84 (79–91)	30	77 (72–88)	13	90 (84–97)	0.011
MAP (mmHg)	45	103 (97.50–109.00)	30	100.50 (94.50–110.25)	13	109.00 (101.50–115.00)	0.28
PP (mmHg)	45	54 (47–71)	30	64 (55–78)^†^^‡^	13	54 (48–62)	0.014
ABI	45	1.15 (1.10–1.20)	30	1.27 (1.19–1.33) ^†^^‡^	13	1.13 (1.10–1.19)	<0.001
ETc (ms)	43	0.367 (0.329–0.402)^†^	29	0.337 (0.311–0.385)	12	0.335 (0.306–0.356)	0.025
B-UT (ms)	43	213 (197–231)	29	193 (152–231)	12	207 (159–246)	0.13
A-UT (ms)	43	176 (163–188)^*^	29	131 (119–159)	12	151 (145–171)	<0.001
Vmax (m/s)	43	4.5 (4.1–4.8)^*^^†^	10	1.8 (1.6–2.1)	5	1.6 (1.3–2.3)	<0.001
EF <50%	45	4 (9%)	30	3 (10%)	13	0 (0%)	0.75

*Pre-operative hemodynamic and stiffness parameters. P-values from analysis of variance (ANOVA), Kruskal-Wallis or Fisher's Exact test as appropriate. HR, heart rate; BP, blood pressure; MAP, mean arterial pressure; PP, pulse pressure; ABI, ankle brachial index; PWV, pulse wave velocity; CAVI, cardio ankle vascular index; baPWV, brachial-ankle PWV; EF, left ventricular ejection fraction; ETc, heart rate corrected ejection time; B-UT, brachial upstroke time; A-UT, ankle upstroke time; Vmax, peak transaortic jet velocity; EF, left ventricular ejection fraction*.

* *Indicate Bonferroni adjusted p-value < 0.05 compared with AR-group*.

† *Indicate Bonferroni adjusted p-value < 0.05 compared with AAD-group*.

‡ *indicate Bonferroni adjusted p-value < 0.05 compared with AVS-group*.

Comparing the groups after adjustment for age, sex, height, MAP, HR, diabetes, eGFR, and CRP revealed that diagnosis group as a significant covariate (*P* 0.005, partial eta^2^ = 0.131; Supplementary Material 4). In the adjusted analysis, AVS and AR patients, displayed lower CAVI compared with the AAD group, with an estimated marginal mean CAVI of 7.60, 7.78, 8.93, for AVS, AR, and AAD, respectively (*p* = 0.005). In the *post-hoc* analysis, both AVS and AR had significantly lower adjusted CAVI compared to AAD (*p* = 0.005 and *p* = 0.015, respectively). In contrast, pre-operative cfPWV did not significantly differ between the groups (*p* = 0.174). Pre-operative baPWV remained significantly higher in the AAD group after adjustments (data not shown).

### Post-operative Assessments

In the post-operative examination, the differences between the groups were attenuated, without significant differences detected for any of the measured parameters (Table 3). No significant difference in cfPWV (*p* = 0.32) or CAVI (*p* = 0.169) were observed between the groups in the adjusted analysis (Supplementary Material 5).

**Table 3:** Post-operative hemodynamic and stiffness parameters.

	**Aortic stenosis**		**Aortic regurgitation**		**Ascending aortic dilatation**		
	***N***	**Median (IQRs) or No. (%)**	***N***	**Median (IQRs) or No. (%)**	***N***	**Median (IQRs) or No. (%)**	***p*-value**
HR (bpm)	32	83 (73–92)	27	84 (76–98)	9	80 (74–87)	0.47
Systolic BP (mmHg)	32	134 (119–145)	27	132 (115–142)	9	128 (116–147)	0.58
Diastolic BP (mmHg)	32	76 (67–81)	27	76 (71–85)	9	77 (67–90)	0.71
MAP (mmHg)	32	96 (89–102)	27	91 (87–104)	9	92 (82–109)	0.94
PP (mmHg)	32	55 (50–65)	27	53 (42–61)	9	51 (46–60)	0.18
ABI	32	1.15 (1.07–1.19)	27	1.20 (1.13–1.26)	9	1.17 (1.00–1.18)	0.06
cfPWV (m/s)	32	8.2 (6.8–9.0)	27	7 (6.3–8.4)	9	8.3 (5.6–9.8)	0.25
CAVI	32	9.13 (8.07–9.55)	27	8.05 (7.22–9.16)	9	8.90 (6.07–11.06)	0.090
baPWV (cm/s)	31	1,441 (1,275–1,586)	27	1,279 (1,189–1,491)	9	1,246 (1,018–1,889)	0.22
ETc (ms)	31	0.336 (0.311–0.376)	27	0.340 (0.318–0.382)	9	0.373 (0.354–0.398)	0.096
B-UT (ms)	31	123 (111–157)	27	127 (104–172)	9	171 (108–179)	0.56
A-UT (ms)	31	123 (115–139)	27	117 (112–128)	9	129 (122–136)	0.12
EF <50%	32	2 (6%)	27	11 (41%)	9	0 (0%)	0.002

*Post-operative measures with comparisons between AVS, AR, and AAD groups with analysis of variance (ANOVA), Kruskal-Wallis or Fisher's Exact test as appropriate. HR, heart rate; BP, blood pressure; MAP, mean arterial pressure; PP, pulse pressure; ABI, ankle brachial index; PWV, pulse wave velocity; CAVI, cardio ankle vascular index; baPWV, brachial-ankle PWV; ETc, heart rate corrected ejection time; B-UT, brachial upstroke time; A-UT, ankle upstroke time; EF, left ventricular ejection fraction*.

### Hemodynamic Changes After Surgery

In the 68 subjects in which both pre- and post-operative measures were complete (Supplementary Figure 1), a paired analysis showed an increase in HR (mean difference 18, SD 13; *p* = < 0.001) and a decrease in MAP (mean difference −6.9, SD 12.7; *p* = < 0.001) on the third post-operative day. Only the AR group displayed significantly decreased systolic blood pressure (SBP) post-operatively, with consequent decreases in pulse pressure and ankle brachial index (Table 4). Ankle upstroke time (A-UT) was significantly prolonged in the AVS compared with AR group at baseline (Table 2) and was diminished after AVR (Table 3) whereas a significant decrease in both A-UT and brachial upstroke time (B-UT) after cardiac surgery was noted in all groups where AVS patients had the largest numerically observed decrease (Table 4). Pre-operative corrected ejection time (ETc) was significantly longer in AVS subjects compared to AAD and borderline compared to AR subjects (Table 2). Post-operatively, there was no significant difference in ETc between the groups and the absolute numbers were inverse to pre-operative, AVR with shortest ETc and AAD longest ETc (Table 3). This was accompanied by a larger decrease in ETc post-operatively in AVS and AR compared with AAD (Table 4). The overall proportion of patients with EF <50 was not significantly different pre-operatively (10%, *n* = 88) and post-operatively (19%, *n* = 68; *P* = 0.17).

**Table 4:** Change in hemodynamic and arterial stiffness parameters after surgery.

	**Aortic stenosis**			**Aortic regurgitation**			**Ascending aortic dilatation**			
	***N***	**Median (IQRs) or mean (SD)**	**Paired *p*-value**	***N***	**Median (IQRs) or mean (SD)**	**Paired *p*-value**	***N***	**Median (IQRs) or mean (SD)**	**Paired *p*-value**	***P*-value**
Δ HR (bpm)	32	14 (8–20)	<0.001	27	19 (9–32)	<0.001	9	21 (7–27)	0.015	0.23
Δ SBP (mmHg)	32	−2 (21)^*^	0.64	27	−17 (24)	0.001	9	−9 (15)	0.11	0.029
Δ DBP (mmHg)	32	−5 (−14 to −1)	0.001	27	−5 (−12 to 5)	0.18	9	−9 (−16 to −1)	0.028	0.39
Δ MAP (mmHg)	32	−6 (12)	0.016	27	−8 (14)	0.009	9	−9 (11)	0.036	0.55
Δ PP (mmHg)	32	3 (−9 to 17)^*^	0.14	27	−14 (−31 to 1)	0.002	9	−4 (−8 to 11)	0.77	<0.001
Δ ABI	32	−0.04 (−0.11 to 0.04)	0.074	27	−0.090 (−0.18 to 0.02)	0.003	9	0.01 (−0.12 to 0.11)	0.95	0.14
Δ baPWV (m/s)	30	155.3 (−1.4 to 303.7)	<0.001	26	126.6 (−39.5 to 213.1)	0.014	8	−83.9 (−228.8 to 232.8)	0.67	0.091
Δ ETc (ms)	30	−0.023 (−0.077 to 0.0074)^†^	<0.001	26	−0.0032 (−0.065 to 0.053)	<0.001	8	0.051 (−0.0079 to 0.063)	0.017	0.006
Δ B-UT (ms)	30	−80 (−108 to −51)	<0.001	26	−59 (−93 to −19)	<0.001	8	−46 (−80 to −20)	0.012	0.077
Δ A-UT (ms)	30	−44 (−56 to −33)^*^	<0.001	26	−18 (−35 to −2)	<0.001	8	−28 (−44 to −13)	0.012	<0.001

*Pairwise comparisons of post-surgery measurement subtracted by baseline measurement (Δ). Data is presented as mean (SD) if normality was proven with Shapiro-Wilk test and absence of outliers. Pairwise comparisons were assessed with paired t-test and Wilcoxon signed rank test as appropriate and comparison of the Δvalue between the three groups was assessed with analysis of variance (ANOVA) or Kruskal-Wallis test as appropriate. HR, heart rate; SBP, systolic blood pressure; DBP, diastolic blood pressure; MAP, mean arterial pressure; ABI, ankle brachial index; baPWV, brachial ankle pulse wave velocity; ETc, heart rate corrected ejection time; B-UT, brachial upstroke time; A-UT, ankle upstroke time*.

* *Indicate Bonferroni adjusted p-value < 0.05 compared with AR-group*.

† *Indicate Bonferroni adjusted p-value < 0.05 compared with AAD-group*.

### Changes in Arterial and Aortic Stiffness After Surgery

**Figure 1:**
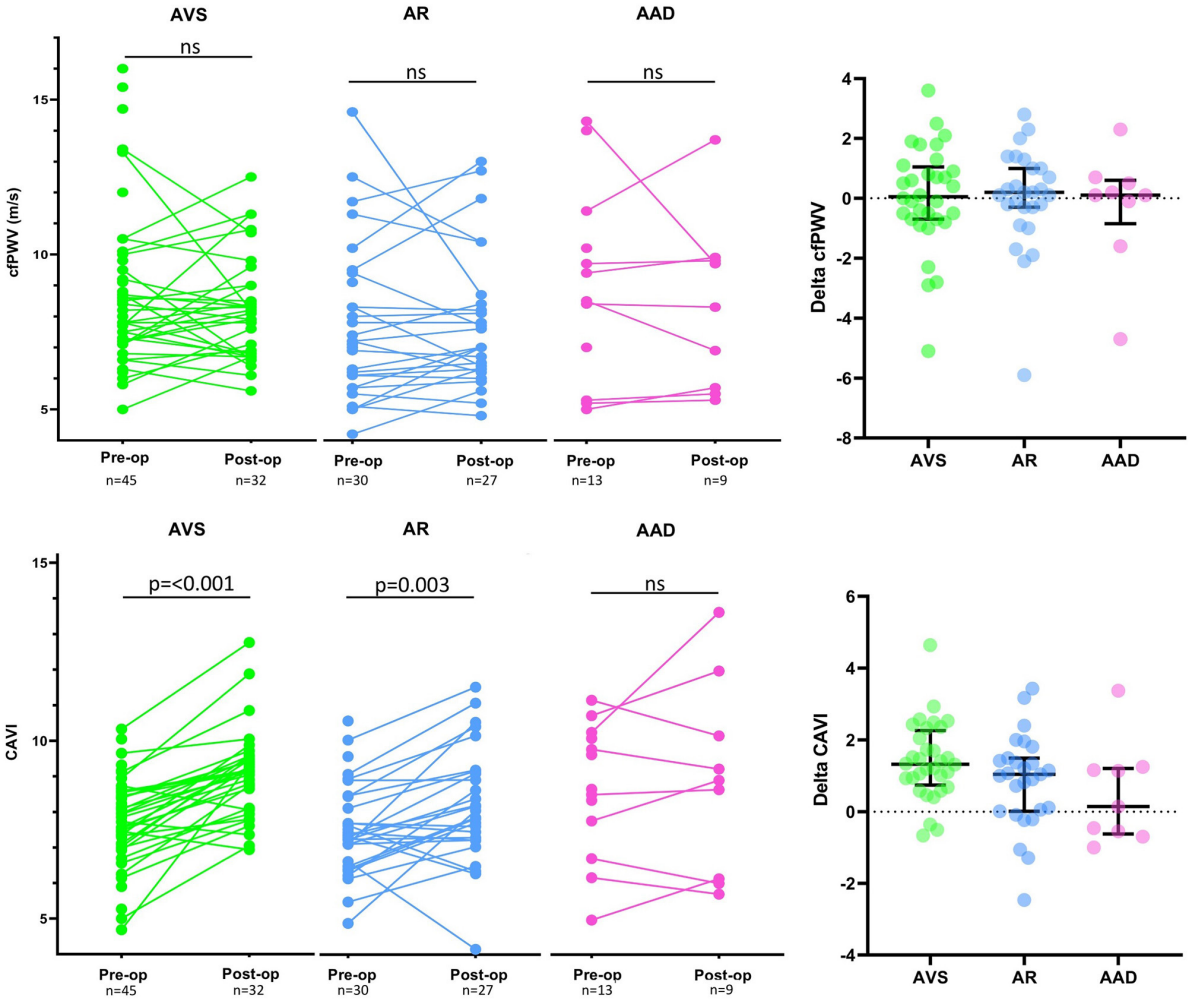
Carotid-femoral pulse wave velocity and cardio ankle vascular index before and after surgery. Graphical presentation of paired measurements in aortic valve stenosis (AVS) patients, aortic regurgitation (AR) patients and ascending aortic dilatation (AAD) patients. The top panel display change in carotid-femoral pulse wave velocity (cfPWV) in respective diagnose group and the right-hand upper panel display the ΔcfPWV (post-surgery cfPWV—baseline cfPWV) for each patient where bars representing median change with interquartile range (IQR). The bottom panel display CAVI measurements in respective diagnose group and the right-hand lower panel display the ΔCAVI (post-surgery CAVI—baseline CAVI) for each patient where bars representing median change with interquartile range (IQR). The comparison between baseline and post-operative measurements was carried out with Wilcoxon signed rank test and ns denotes not significant.

CAVI increased significantly after surgery in patients with AVS (median 1.33, IQR 0.74–2.26; *p*-value < 0.001) and AR (median 1.04, IQR 0.01–1.49; *p*-value 0.003) whereas no significant change was observed in the AAD group (median 0.14, IQR −0.63 to 1.21; *p*-value 0.31), depicted in Figure 1. The observed increase in peripheral arterial stiffness was numerically higher in AVS subjects compared to AR subjects (Figure 1). In line with the observed changes in CAVI, baPWV increased significantly in both AVS and AR (Table 4). In contrast, cfPWV did not significantly change after surgery in AVS (median 0.05, IQR −0.7 to 1.1; *p*-value 0.62), AR (median 0.2, IQR −0.3 to 1.0; *p*-value 0.41) nor AAD (median 0.1, IQR −0.9 to 0.6; *p*-value 0.64), see Figure 1. A repeated measures ANCOVA adjusting for pre- and post-operative differences in MAP and HR did not affect the results (data not shown). The between groups comparisons were not significantly different for any of the stiffness parameters.

### Predictors of Post-operative Change in CAVI

**Figure 2:**
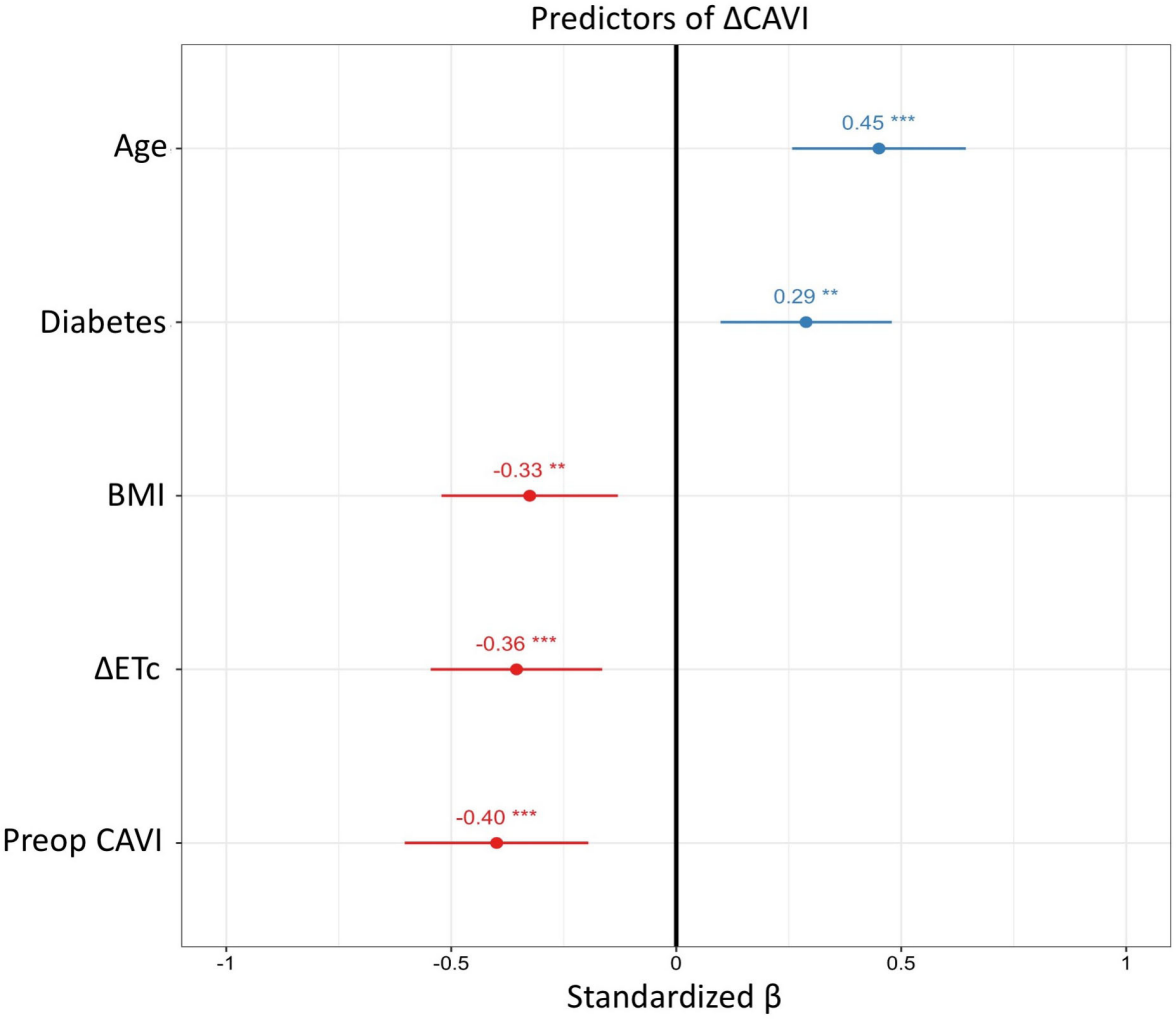
Predictors of change in cardio ankle vascular index. Results from the multivariate linear regression including AVS and AR patients with baseline and post-surgery measurements (*n* = 56). Round dots represent standardized β coefficient in with 95% confidence intervals to make the predictors comparable regardless of scale. **p-value < 0.01, and ***p-value < 0.001. Δ, post-operative measurement subtracted by baseline measurement; HR, heart rate; ETc, heart rate corrected ejection time; CAVI, cardio ankle vascular index; BMI, body mass index.

In patients undergoing AVR (AVS and AR groups, *n* = 56), independent predictors for the increase in CAVI were determined by a stepwise multivariate model using backwards selection and including ETc, CVD risk factors and hemodynamic factors such as MAP. Age, diabetes, BMI, ΔETc and pre-operative CAVI were retained in the model, yielding an *R*^2^ = 0.61 (Figure 2). Age, low pre-operative CAVI and decreased ETc were the most prominent predictors of increased CAVI after surgery.

## Discussion

The results of the present study identify three main novel aspects of aortic valvulo-vascular interactions and peripheral arterial stiffness. First, we show that pre-operative arterial stiffness was lower in patients with aortic valve- compared with aortic pathologies, and that this difference disappeared after surgery. Second, CAVI increased after aortic valve but not isolated aortic surgery, in particular in AVS patients. Third, changes in CAVI after aortic valve surgery were associated with ETc, age, pre-operative CAVI, BMI, and diabetes in a multivariate regression model explaining over 60 % of the variation. Taken together, these observations indicate that both CV risk factors and hemodynamic alterations affect arterial stiffness in aortic valve disease.

The lower arterial stiffness in patients with aortic valve disease compared to younger patients in the AAD group was counterintuitive. This difference was equivalent to a 5 years' younger vascular age than expected in patients with AVS as estimated by CAVI. However, the significant increase in CAVI after AVR resulted in a 10 years' increase in vascular age, indicating that significant arterial stiffness may be masked by hemodynamic consequences of AVS such as prolonged ET. Likewise, cardiovascular risk factors were more strongly associated with CAVI in the post-operative compared with the pre-operative cohorts, further supporting that correction of the aortic valvulopathy enables an adequate arterial stiffness evaluation. Importantly, PWV in AAD-patients is not dependent on the underlying pathology since PWV in BAV did not differ from degenerative ascending aortic aneurysms in TAV (28). In contrast, aortic stiffness in Marfan syndrome is greater compared with BAV patients with similar aortic sizes (28). The inclusion of only one Marfan patient in the AAD group in the present study did not allow a comparative analysis but when excluded from the paired analyses, did not influence the change in stiffness parameters (data not shown) and is unlikely to affect other results to a great extent. Whereas, some previous studies showed increased stiffness measures after AVR for AVS (9–11, 14, 15), others reported either no change (11, 13, 16) or even a decrease (12, 29, 30). In the interpretation of the variable results in those studies, it should also be considered that the methods used have different degrees of validation for the evaluation of arterial stiffness with and without AVS. The present study extends those previous findings by being first to report increased CAVI and baPWV after surgical AVR for AVS. We also observed an increased CAVI after AVR for AR, suggesting that a masked arterial stiffness would be applicable to aortic valve disease in general, although the increase in CAVI after AVR was most pronounced in the AVS compared with the AR group. In contrast, isolated aortic surgery did not significantly alter arterial stiffness, arguing against that thoracic surgery *per se* was the driving factor behind the observed changes in arterial stiffness.

In contrast to CAVI, we did not detect any significant differences in cfPWV either between the diagnosis groups or before and after surgery in this study. Previous studies of changes in cfPWV after aortic valve surgery are inconsistent with increased (14, 15) as well as unchanged (16) PWV reported after SAVR and TAVR. The observed decrease in MAP post-operatively may have compensated an increased cfPWV following AVR. However, since baPWV, which has similar BP dependence as cfPWV, significantly increased after AVR, it is unlikely that blood pressure changes would explain the absence of changed cfPWV following SAVR in the present study. Furthermore, adjustment for the change in HR and MAP did not reveal any post-operative change in cfPWV. It is important to consider that cfPWV mainly measure stiffness of the aorta, whereas CAVI includes distal smaller and less compliant muscular arteries (31–33) with a gradual increase in PWV from the ascending aorta to the iliac artery (34). The change in peripheral arterial stiffness in the present study (CAVI and baPWV) suggests that a potential masking effect of aortic valvulopathies on arterial stiffness would be more likely detected when intrinsically stiffer arteries are included. This notion is further supported by a previous study reporting a larger increase in baPWV compared to cfPWV after TAVI (14). However, this also implies that an increased cfPWV may be detected using more patients, albeit of smaller magnitude compared to CAVI. Lastly, ventriculo-arterial coupling (VAC) normalize after AVR (35) and is more associated with measures of peripheral derived arterial stiffness compared to cfPWV (36). Hence, CAVI and baPWV might better reflect the change in VAC following AVR.

Multivariate analysis identified both baseline CV risk factors and changes in ETc as predictors of an increased CAVI following AVR. Whereas, age and diabetes, known predictors of arterial stiffness (19), as well as low pre-operative CAVI were associated with a more pronounced increase in CAVI after AVR, higher BMI was associated with a less pronounced increase in post-operative CAVI. The inverse association for CAVI with BMI was significant in the post-operative examinations in the present study, which has also been previously reported for indices of obesity in previous studies (19, 37). Independently of these baseline characteristics, the increase in CAVI after AVR was associated with the concomitant decrease in ETc. Indeed, the classical clinical *pulsus parvus et tardus* (weak and prolonged pulse) in aortic valve disease is caused by an increased ET. The resulting prolonged pulse wave leads to an extended and larger arterial dilatation to comply with the systolic flow and consequently resulting in decreased measures of arterial stiffness. The results of the present study implicate that by normalizing the ET through AVR, the arterial stiffness can appropriately be captured resulting in an increased CAVI. The increased CAVI observed in AVSc patients without significantly affected hemodynamics support this hypothesis (5).

The clinical importance of arterial stiffness in AVS patients has been established through the prognostic value of valvulo-arterial impedance (Zva), which measures the combined load on the LV exerted by the arterial tree and the stenotic aortic valve (7). Furthermore, pre-operative cfPWV is associated with heart failure, cognitive dysfunction and poor quality of life in AVS (38–40). However, the clinical importance of changes in arterial stiffness after AVR remains to be elucidated. The underestimated pre-operative arterial stiffness, which is suggested from the results of the present study, raises the notion to consider the ET when evaluating arterial stiffness in aortic valve disease. Likewise, the prognostic value of post-operative CAVI warrant further exploration as peripheral arterial stiffness has not been as extensively studied in terms of outcome.

The repeated pre- and post-operative measures by the same investigator in the same setting to minimize inter-examination variations, and the rigorous adjustments for changes after AVR are methodological strengths of this study. Certain limitations should however be acknowledged. First, 23% of the patients did not undergo post-operative assessment and hence there were relatively few patients available for pre- and post-operative measures.

Atrial fibrillation was a more common post-operative exclusion criteria in the AR group but similar between AVS and AAD groups and hence unlikely to bias the results. Second, the effects on arterial stiffness after cardiac surgeries other than for AVS, AR, and AAD were not examined. Third, combined valvulopathies and/or AAD were not among the exclusion criteria in this study. However, given the minimal overlap between the diagnosis groups, this is expected to have negligible impact on the observed results. Forth, technical limitations of measuring CAVI in AVS and AR have previously been raised both by researchers (41) and manufacturers, due to the phonocardiogram used to time the cardiac cycle may be hampered by murmurs caused by AVS and AR. However, only 4 patients (<4%) were excluded due to poor registration of CAVI and only measurements deem adequate by the device were included in the present study. Also, the prolonged pulse wave observed in AVS was not subject to technical concern since the foot of the pulse wave is used for analysis. The automated oscillometric blood pressure measurement may represent an additional technical limitation in valvular heart disease, in particular aortic regurgitation. Fifth, there was a slight overlap of AAD between the groups which could be a potential confounder. The study was not designed to provide coefficient of variation for the devices although previous studies reported 2.4% inter-observer variability for CAVI with Vasera device (21) which is lower compared to 9% reported for cfPWV with Sphygmocor (42). The variability should not impact the paired results of the present study due to the large detected difference. We did not provide pre- and post-operative echocardiographic hemodynamics. These conditions lead to the limitation of the present study that complete adjustment was not able to be performed for all possible confounding factors. However, the most plausible confounders have been taken into account by controlling for the change in MAP and HR while other covariates are inherently adjusted for by the repeated measures design. Last, while the low proportion of study participants with decreased EF limits the extrapolation of the present results to heart failure, cfPWV remains a predictive stiffness measure independent of EF (43).

In summary, the results of the present study indicate that arterial stiffness may be underestimated in AVS and AR, and that the post-operative stiffness better reflects the patient's true vascular status. In particular, cardiovascular risk factors and baseline CAVI along with changes in ET predicted an increased CAVI after AVR. Since significant arterial stiffness impacts valvulo-arterial coupling, the interpretation of arterial function may contribute to the evaluation of LV load and prognosis in aortic valve disease.

## Data Availability Statement

Individual participant data that underlie the results reported will be shared, after deidentification, with researchers who provide a methodologically sound proposal.

## Ethics Statement

The study was reviewed and approved by Regionala Etikprövningsnämnden I Stockholm (2012/1633-31/4, amendment 2016/2346-32). The patients/participants provided their written informed consent to participate in this study.

## Author Contributions

OP, AF-C, and MB: conception and design of the work, interpretation, draft of manuscript, and substantial revision. OP: acquisition. OP and MB: analysis. All authors made substantial contribution to the manuscript.

## Funding

This work was supported by the Swedish Research Council (grant number 2019-01486), the Swedish Heart and Lung Foundation (grant number 20180571), the King Gustaf V and Queen Victoria Freemason Foundation, and Region Stockholm County Council (grant number 20170365). OP was supported by the Clinical Scientist Training Programme (CSTP) at Karolinska Institute. AF-C was supported by a donation from Mr. Fredrik Lundberg.

## Conflict of Interest

The authors declare that the research was conducted in the absence of any commercial or financial relationships that could be construed as a potential conflict of interest. The reviewer TK declared a shared affiliation with several of the authors, OP, AF-C, and MB, and to the handling editor at time of review.

## Publisher's Note

All claims expressed in this article are solely those of the authors and do not necessarily represent those of their affiliated organizations, or those of the publisher, the editors and the reviewers. Any product that may be evaluated in this article, or claim that may be made by its manufacturer, is not guaranteed or endorsed by the publisher.
