# Supplementary material for: Cardiovascular Risk Factors and Hemodynamic Measures as Determinants of Increased Arterial Stiffness Following Surgical Aortic Valve Replacement
Source: Front Cardiovasc Med. 2021 Dec 8;8:754371. doi: 10.3389/fcvm.2021.754371 (PMC8692982; doi:10.3389/fcvm.2021.754371)
Supplement: Supplementary file 1 [file Data_Sheet_1.PDF]

Supplementary Material

Supplement 1 – Flow Chart of Included and Excluded Patients in the Study

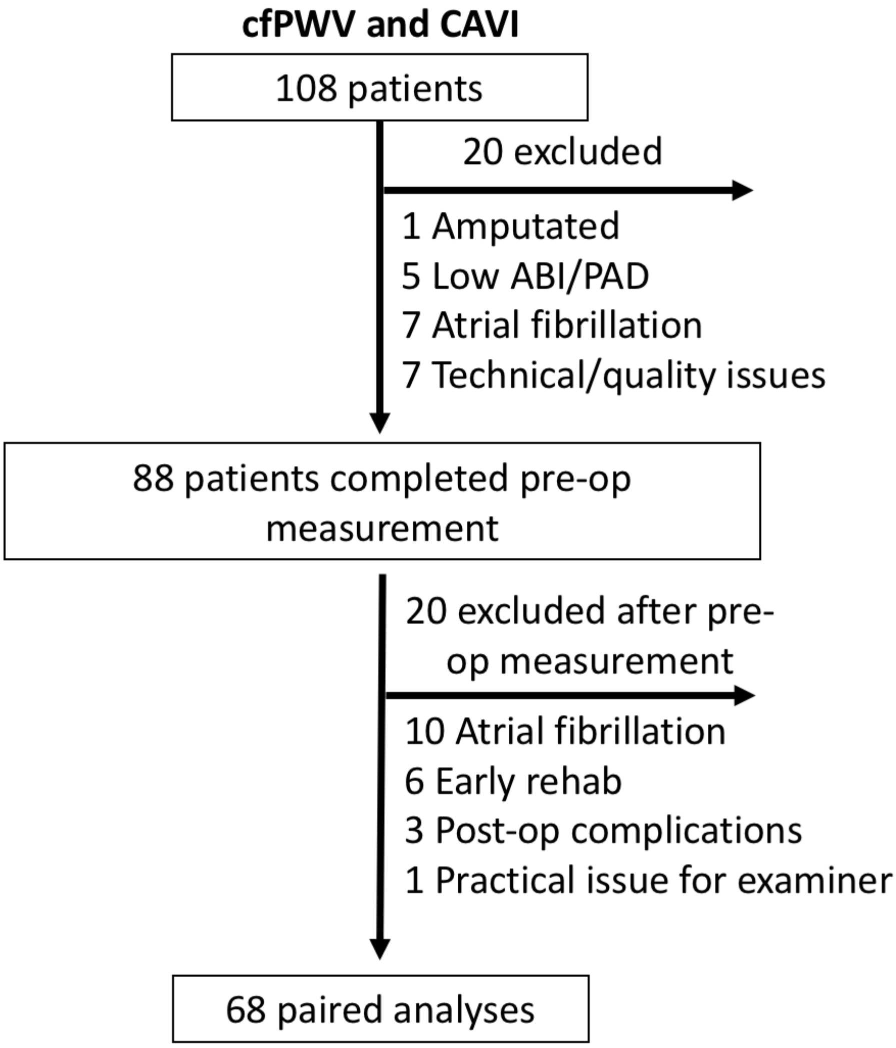

Flowchart of patients starting from patients approach for inclusion in the study.

Supplement 2 - Univariate correlations of markers of stiffness, clinical characteristics and hemodynamic parameters

	CAVI 1	CAVI 2	Δ CAVI	PWV 1	PWV 2	Δ PWV
Pre CAVI, n=88	1	0.684‡	-0.175	0.484‡	0.580‡	-0.006
Post CAVI, n=68	0.684‡	1	0.598‡	0.539‡	0.703‡	0.099
Delta CAVI, n=68	-0.175	0.598‡	1	0.169	0.312*	0.140
PWV_1 (m/s), n=88	0.484‡	0.539‡	0.169	1	0.707‡	-0.533‡
PWV_2 (m/s), n=68	0.580‡	0.703‡	0.312*	0.707‡	1	0.221
Delta PWV (m/s), n=68	-0.006	0.099	0.140	-0.533‡	0.221	1
Age (years)	0.360†	0.599‡	0.448‡	0.482‡	0.538‡	-0.024
Male sex	0.002	-0.128	-0.168	-0.318†	-0.235	0.087
BMI (kg/m^2)	-0.102	-0.303*	-0.123	0.141	-0.094	-0.216
MAP (mmHg)	0.226*	0.002	-0.179	0.408‡	0.107	-0.445‡
MAP_2 (mmHg)	0.198	0.176	0.020	-0.021	0.159	0.220
Delta MAP (mmHg)	0.052	0.167	0.168	-0.365†	0.064	0.580‡
HR (bpm)	0.126	0.210	0.244*	0.163	0.339†	0.066
HR_2 (bpm)	-0.023	-0.261*	-0.327†	0.099	0.014	-0.120
Δ HR (bpm)	-0.040	-0.339†	-0.415‡	-0.048	-0.173	-0.140
AVR	-0.213*	0.071	0.315†	-0.066	-0.043	0.059
CAD	0.080	0.232	0.150	0.113	0.014	-0.086
AVS	-0.044	0.199	0.260*	0.133	0.095	0.026
AR	-0.199	-0.248*	-0.134	-0.237*	-0.189	-0.053

Hypertension	0.201	0.239	0.057	0.215*	0.135	-0.279*
Diabetes	0.067	0.251*	0.229	0.199	0.215	0.056
eGFR (ml/min/m^2)	-0.211*	-0.197	-0.147	-0.229*	-0.127	0.051
CRP (mg(dL)	0.162	0.212	0.100	0.224*	0.368†	0.081

Univariate Pearson correlations between cardio ankle vascular index (CAVI), carotid-femoral pulse wave velocity (cfPWV) and clinical and hemodynamic parameters. BMI=body mass index, MAP=mean arterial pressure, HR=heart rate, AVR=aortic valve replacement, AR=aortic regurgitation, CAD=coronary artery disease, AVS=aortic valve stenosis, AR=aortic regurgitation, eGFR=estimated glomerular filtration rate, CRP=C-reactive protein. _2 after the variable denotes post-operative measurement. * p-value <0.05, † p-value <0.01, ‡ p-value <0.001

Supplement 3 - Univariate correlations of brachial ankle pulse wave velocity, clinical characteristics and hemodynamic parameters

	baPWV_1	baPWV_2	ΔbaPWV
baPWV_1 (cm/s), n=84	1	0.715†	-0.019
baPWV_2 (cm/s), n=67	0.715†	1	0.685†
ΔbaPWV (cm/s), n=64	-0.019	0.685†	1
PWV_1 (m/s)	0.656†	0.596†	0.075
PWV_2 (m/s)	0.672†	0.779†	0.430†
ΔPWV (m/s)	-0.208	0.108	0.413†
CAVI_1	0.777†	0.654†	0.159
CAVI_2	0.651†	0.839†	0.514†
ΔCAVI	0.054	0.414†	0.518†
Age (years)	0.472†	0.509†	0.222
Male sex	-0.133	-0.192	-0.153
BMI (kg/m^2)	0.037	-0.172	-0.209
MAP (mmHg)	0.486†	0.136	-0.284*
MAP_2 (mmHg)	0.197	0.368†	0.291*
ΔMAP (mmHg)	-0.142	0.238	0.503†
HR (bpm)	0.062	0.195	0.198
HR_2 (bpm)	-0.002	0.051	0.062
ΔHR (bpm)	-0.032	-0.062	-0.050
AVR	-0.342†	0.049	0.336†

CAD	0.074	0.124	-0.002
AVS	-0.083	0.167	0.271*
AR	-0.237*	-0.201	-0.113
Hypertension	0.320†	0.322†	0.017
Diabetes	0.083	0.237	0.199
eGFR (ml/min/m^2)	-0.358†	-0.196	0.043
CRP (mg/dL)	0.169	0.209	0.136

Univariate Pearson correlations between brachial ankle pulse wave velocity (baPWV), clinical and hemodynamic parameters. BMI=body mass index, MAP=mean arterial pressure, HR=heart rate, AVR=aortic valve replacement, AR=aortic regurgitation, CAD=coronary artery disease, AVS=aortic valve stenosis, eGFR=estimated glomerular filtration rate, CRP=c-reactive protein. _2 after the variable denotes post-operative measurement. Δ=post-operative measure – pre-operative measure. * p-value<0.05, † p-value <0.001.

Supplement 4 - Adjusted Baseline CAVI and cfPWV in Aortic Stenosis, Aortic Regurgitation and Ascending Aortic Dilation patients

	Baseline CAVI, n=87			Baseline cfPWV, n=85		
Source	F	p-value	Partial Eta Squared	F	p-value	Partial Eta Squared
Corrected Model	4.138	<0.001	0.353	9.057	0.000	0.550
Intercept	0.837	0.363	0.011	3.642	0.060	0.047
Age (years)	15.991	<0.001	0.174	32.122	0.000	0.303
Sex	0.001	0.970	<0.001	4.274	0.042	0.055
Height (cm)	2.359	0.129	0.030	4.408	0.039	0.056
MAP (mmHg)	1.647	0.203	0.021	20.405	<0.001	0.216
HR (bpm)	2.473	0.120	0.032	2.389	0.126	0.031
Diabetes	0.380	0.540	0.005	2.488	0.119	0.033
eGFR (ml/min/1.73m2)	0.192	0.663	0.003	2.489	0.119	0.033
CRP <1 mg/dL	4.743	0.033	0.059	1.687	0.198	0.022
Diagnosis group (AVS/AR/AAD)	5.719	0.005	0.131	1.792	0.174	0.046

Table displaying results from two analysis of covariance (ANCOVA) with CAVI (cardio ankle vascular index) and cfPWV (carotid-femoral pulse wave velocity) as dependent variables and diagnose group as fixed factor. The covariates were chosen based on clinical reasoning and univariate Pearson correlation. Appropriate ANCOVA assumptions were met after excluding 1 outlier (low) from the AVS group in the CAVI analysis and 3 AVS outliers (high) in the cfPWV followed by log2 transformation. MAP=mean arterial pressure, HR=heart rate, eGFR=estimated glomerular filtration rate, CRP=c-reactive protein, AVS=aortic valve stenosis, AR=aortic regurgitation, AAD=ascending aortic dilatation. Partial eta is an indicator of effect size.

Supplement 5 - Adjusted post-operative CAVI and cfPWV in Aortic Valve Stenosis, Aortic Regurgitation and Ascending Aortic Dilation patients

	Post-operative CAVI, n=66			Post-operative cfPWV, n=66		
Source	F	p-value	Partial Eta Squared	F	p-value	Partial Eta Squared
Corrected Model	7.583	<0.001	0.580	6.212	<0.001	0.530
Intercept	4.926	0.031	0.082	0.051	0.822	0.001
Age (years)	49.215	<0.001	0.472	23.167	<0.001	0.296
Sex	1.088	0.302	0.019	5.924	0.018	0.097
Height (cm)	.135	0.714	0.002	1.374	0.246	0.024
Diabetes	1.339	0.252	0.024	6.320	0.015	0.103
eGFR (ml/min/1.73m2)	4.154	0.046	0.070	2.490	0.120	0.043
CRP <1	.121	0.729	0.002	3.235	0.078	0.056
Postop MAP (mmHg)	6.879	0.011	0.111	4.885	0.031	0.082
Postop HR (bpm)	2.120	0.151	0.037	0.291	0.592	0.005
Diagnosis group	1.838	0.169	0.063	1.163	0.320	0.041

Table displaying results from two analysis of covariance (ANCOVA) with CAVI (cardio ankle vascular index) and cfPWV (carotid-femoral pulse wave velocity) as dependent variables and diagnose group as fixed factor. The covariates were chosen based on clinical reasoning and univariate Pearson correlation. Appropriate ANCOVA assumptions were met after log2-transformation and excluding 1AVS outlier (high) and 1 AR outlier (low) in the CAVI analysis and 2 AR cfPWV outliers (high). MAP=mean arterial pressure, HR=heart rate, eGFR=estimated glomerular filtration rate, CRP=c-reactive protein, AVS=aortic valve stenosis, AR=aortic regurgitation, AAD=ascending aortic dilatation. Partial eta is an indicator of effect size.
